# Clinically silent *Plasmodium vivax* infections in native Amazonians of northwestern Brazil: acquired immunity or low parasite virulence?

**DOI:** 10.1590/0074-02760220175

**Published:** 2022-12-16

**Authors:** Luiza Barbosa Barros, Priscila Rodrigues Calil, Priscila Thihara Rodrigues, Juliana Tonini, Pablo Secato Fontoura, Priscila Moraes Sato, Marly Augusto Cardoso, Marina Werneck de Almeida Avellar Russo, Carlos Eduardo Cavasini, Anderson Rocha de Jesus Fernandes, Marcelo Urbano Ferreira

**Affiliations:** 1 Universidade de São Paulo, Instituto de Ciências Biomédicas, Departamento de Parasitologia, São Paulo, SP, Brasil; 2 Universidade de São Paulo, Faculdade de Saúde Pública, Departamento de Nutrição, São Paulo, SP, Brasil; 3 Faculdade de Medicina de São José do Rio Preto, Departamento de Doenças Dermatológicas, Infecciosas e Parasitárias, São José do Rio Preto, SP, Brasil; 4 Universidade Nova de Lisboa, Instituto de Higiene e Medicina Tropical, Lisboa, Portugal

**Keywords:** malaria, Plasmodium vivax, asymptomatic infections, Amazon, molecular diagnosis

## Abstract

**BACKGROUND:**

Malaria remains common among native Amazonians, challenging Brazil′s elimination efforts.

**OBJECTIVES:**

We examined the epidemiology of malaria in riverine populations of the country′s main hotspot - the upper Juruá Valley in Acre state, close to the Brazil-Peru border, where *Plasmodium vivax* accounts for > 80% of cases.

**METHODS:**

Participants (n = 262) from 10 villages along the Azul River were screened for malaria parasites by microscopy and genus-specific, *cytochrome b* (*cytb*) gene-based polymerase chain reaction. Positive samples were further tested with quantitative TaqMan assays targeting *P. vivax-* and *P. falciparum*-specific *cytb* domains. We used multiple logistic regression analysis to identify independent correlates of *P. vivax* infection.

**FINDINGS:**

Microscopy detected only one *P. vivax* and two *P. falciparum* infections*.* TaqMan assays detected 33 *P. vivax* infections (prevalence, 11.1%), 78.1% of which asymptomatic, with a median parasitaemia of 34/mL. Increasing age, male sex and use of insecticide-treated bed nets were significant predictors of elevated *P. vivax* malaria risk. Children and adults were similarly likely to remain asymptomatic once infected.

**MAIN CONCLUSIONS:**

Our findings are at odds with the hypothesis of age-related clinical immunity in native Amazonians. The low virulence of local parasites is suggested as an alternative explanation for subclinical infections in isolated populations.

Malaria transmission has decreased substantially over the past 2 decades in Latin America and the Caribbean,^1^ where 653,000 new cases were notified in 2020.^2^ The Amazon basin accounts for approximately 90% of the malaria cases across the region,^1^ 68% of which due to *Plasmodium vivax*.^2^ With nearly 150,000 notifications in 2020, Brazil alone recorded > 20% of malaria cases in the Americas.^2^ More intense residual transmission is observed in hard-to-reach riverine villages and Amerindian reserves, gold mining enclaves and frontier farming settlements.^3^

Since the early 2000s, traditional populations scattered along the margins of the Amazonian rivers are known to harbour low-density malarial infections, often missed by microscopy and rapid diagnostic tests (i.e., subpatent) but detected by sensitive molecular techniques such as the polymerase chain reaction (PCR).^4^ ^-^ ^8^ These infections are typically asymptomatic, especially among adults - a finding interpreted as evidence of acquired clinical immunity that gradually protects rural Amazonians from high parasitaemia and overt malaria.^4^ ^,^ ^6^ Importantly, asymptomatic *P. vivax* carriers often harbour mature gametocytes,^9^ can experimentally infect local malaria vectors^10^ ^-^ ^12^ and are usually overlooked by routine surveillance.^13^ ^,^ ^14^

Here we show that low-density and asymptomatic *P. vivax* infections remain frequent in isolated riverine villages of the upper Juruá Valley region, a transmission hotspot in northwestern Brazil that contributes nearly 18% of the country′s malaria cases. Adults are not more likely than young children to remain asymptomatic once infected, contrary to the expectations from the age-related clinical immunity hypothesis. We suggest an alternative explanation for the high frequency of subclinical malarial infections in these and similar isolated Amazonian populations.

## SUBJECTS, MATERIALS AND METHODS

*Study area and population -* This study took place in the municipality of Mâncio Lima, in the upper Juruá Valley region of Acre state, westernmost Brazil, close to the border with Peru (Figure). With a typical equatorial humid climate, the area receives most rainfall between November and April, but malaria transmission occurs year-round. *Anopheles* (*Nyssorhynchus*) *darlingi* is the primary vector and *P. vivax* accounts for > 80% of local malaria cases.^15^

**Figure:**
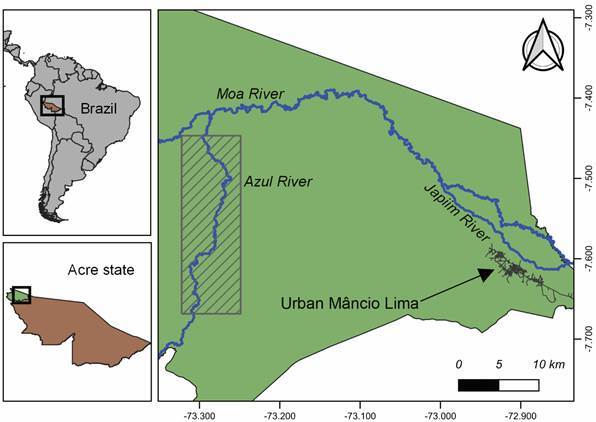
Study site. The upper left panel shows Brazil in South America and the location of Acre state, while the lower left panel shows the municipality of Mâncio Lima (green) in the upper Juruá Valley region. The rural area of Mâncio Lima is shown in greater detail in the right panel. Rivers are represented in blue. Figure prepared by Igor C. Johansen with QGIS software version 3.14. Publicly available shapefiles were obtained from Instituto Brasileiro de Geografia e Estatística (IBGE) website (https://bit.ly/34gMq0S) and river locations from the OpenStreetMap Foundation website (https://bit.ly/3pzh4xp).

We carried out cross-sectional malaria prevalence surveys in 10 riverine villages or “communities” on the banks of the Azul River, a tributary of the Moa River, all of them originated from former rubber-tapper settlements: Três Unidos, Valparaíso, Barro Vermelho, Belo Horizonte, Buritis, Nova Lição, Bom Sossego, Bela Vista, Bom Jesus and Queimadas (Table I). These villages are situated between 6 and 10 h, by motorboat, from the town of Mâncio Lima. No recent population estimates were available for the study sites. 

**TABLE I:** Study sites along the Azul River, upper Juruá Valley, Acre state, Brazil, and population examined in 2018 and 2019

			Number enrolled and positive (tested in 2018)			Number enrolled and positive (tested in 2019)		
Village	Latitude	Longitude	Enrolled	Microscopy	TaqMan	Enrolled	Microscopy	TaqMan
Três Unidos	-7.5114	-73.2699	55	0/37	2/35	43	0/34	5/34
Valparaíso	-7.5361	-73.2762	5	0/2	1/2	0	0/0	0/0
Buritis	-7.5741	-73.2860	23	0/20	2/19	25	0/14	1/13
Belo Horizonte	-7.6198	-73.3226	6	0/6	0/6	3	0/1	0/1
Nova Lição	-7.6429	-73.3064	32	2/23	6/23	38	0/18	0/17
Barro Vermelho	-7.6760	-73.3125	26	0/10	3/10	24	1/7	2/9
Bom Sossego	-7.7292	-73.3498	99	0/50	4/50	64	0/43	6/44
Bela Vista	ND	ND	0	0/0	0/0	12	0/8	0/7
Bom Jesus	-7.7964	-73.3860	0	0/0	0/0	32	0/24	3/24
Queimadas	-7.8518	-73.4072	0	0/0	0/0	3	0/1	0/1
Total (% tested)			246	148 (60.2)	145 (58.9)	244	150 (61.5)	150 (61.5)

ND: not determined.

During the first survey, carried out between 9 and 12 July, 2018 (hereafter “2018 survey”), our field team carried out house-to-house visits in seven villages. We used a structured questionnaire to obtain sociodemographic and morbidity information from 246 individuals aged between 1 and 72 years, who were invited to provide a finger-prick blood sample for malaria diagnosis and haemoglobin measurement using a HemoCue Hb 301 haemoglobinometer (HemoCue AB, Angelholm, Sweden). Anaemia was defined using the age- and sex-specific haemoglobin cut-off levels recommended by the World Health Organization (WHO).^16^ Data on selected household assets were combined to derive a wealth index - a proxy of socioeconomic status.^17^ A total of 148 (60.2%) study participants were screened for malaria parasites by microscopy or PCR in 2018 (Table I). 

During the second survey, in 9-12 July, 2019 (hereafter “2019 survey”), residents in the area were invited to attend medical and dental care, routine vaccination and cervical cancer screening in health posts or schools situated in one of three largest villages (Três Unidos, Nova Lição and Bom Sossego). A total of 244 attendants aged < 1-75 years, coming from nine villages, were interviewed using the same structured questionnaire applied in 2018; 150 (61.5%) participants provided finger-prick capillary blood for malaria diagnosis by microscopy and the same number by molecular methods (Table I). Overall, we enrolled 386 individuals and tested 262 (67.9%) of them for malaria parasites during at least one survey; 36 (9.3%) participants were tested during both surveys.

At enrolment, participants were asked whether they had any clinical sign or symptom that might have been caused by malaria. Participants reporting recent signs or symptoms at or up to 7 days prior to the interview were classified as “symptomatic”. We next applied a semiquantitative questionnaire addressing 13 common symptoms (fever, chills, sweating, headache, myalgia, arthralgia, abdominal pain, nausea, vomiting, dizziness, cough, dyspnoea and diarrhoea) to all symptomatic individuals.^18^ ^)^ According to the individuals’ perception, each clinical manifestation was classified as absent, mild, moderate or severe.

*Laboratory diagnosis of malaria -* A total of 298 thick blood smears were prepared, stained with Giemsa and had at least 100 fields examined for malaria parasites, under 1,000x magnification, by an experienced microscopist.^19^ *P. vivax* infections diagnosed by onsite microscopy were treated with chloroquine (25 mg/kg of body weight over 3 days) and primaquine (3.5 mg/kg over 7 days); *Plasmodium falciparum* infections were treated with a 3-day course of artemether (2 to 4 mg/kg/day) plus lumefantrine (12 to 24 mg/kg/day) and a single dose of 0.25 mg/kg primaquine for gametocyte clearance.^20^

Fifty-millilitre capillary blood samples (n = 295) kept in liquid nitrogen in the field and later stored at -20°C were used for parasite DNA extraction, with QIAsymphony DNA Investigator kits (Qiagen, Hilden, Germany), on an automated QIASymphony platform (Qiagen). The final DNA elution volume was 100 µL. Molecular screening for malaria was carried out with a genus-specific, SYBR Green-based quantitative PCR. The primer pairs (PCBF, 5’-ATG CTT TAT TAT GGA TTG GAT GTC-3’ and PCBR, 5’-CAG ACC GTA AGG TTA TAA TTA TGT-3’) target a conserved sequence of the *cytochrome b* (*cytb*) gene of human-infecting malaria parasites,^21^ with a detection threshold of 0.2 amplicon copies per µL (corresponding to approximately four parasites per mL, assuming an average of 50 mitochondrial genome copies per uninuclear blood-stage parasite). The 20-µL reaction contained 5 µL of DNA solution, 7.5 µL of 2× Maxima SYBR Green quantitative PCR master mixture (Fermentas, Burlington, Canada) and 0.3 µM of each primer. The amplification protocol comprised a 2-min step at 50°C, followed by 10-min denaturation step at 95°C and 40 cycles (95°C for 15 s and 60°C for 1 min) on a QuantStudio 6 real-time PCR system (Thermo Fisher Scientific, Waltham, USA).

Positive samples were further tested with newly designed TaqMan assays that target species-specific *cytb* gene fragments. The *P. vivax* protocol used the primers 5’-TTT GGT GGT ACT ACA GGA GTA ATA TTA GGT-3’ and 5’-GAA ATG AGC GAT TAC ATA GTA AGT ATC ATG-3’ and the probe 5’-VIC-TGC AGC TAT TGA TAT TGC AT-MGB-NFQ-3’ (target fragment size, 148 base pairs [bp]); the *P. falciparum* protocol used the primers 5’-CAT TAT GAT TAC AGC TCC CAA GCA-3’ and 5’-GGT CTG ATT TGT TCC GCT CAA TA-3’ and the probe 5’ FAM -TAC AAG ATT GTG ATA AGA TGA C-NFQ-MGB-3’ (target fragment size, 90 bp). Each 20-µL reaction for both assays, carried out in in 96-well microplates, contained 5 µL of DNA solution and 10 µL of 2 TaqMan Universal Mater Mix II, no-UNG (Thermo Fisher Scientific). In the *P. vivax* protocol, we used 0.2 µM of each primer and 0.1 µM of the probe per reaction; in the *P. falciparum* protocol, we used 0.1 µM of each primer and 0.08 µM of the probe. The amplification cycles were identical for both species and comprised a 2-min step at 50°C followed by a 10-min denaturation at 95°C and 45 cycles at 95°C for 15 s and at 60°C for 1 min, carried out on a QuantStudio 6 real-time PCR system (Thermo Fisher Scientific). Standard curves were prepared with nine serial 10-fold dilutions of the respective target sequences and tested in each microplate to allow for species-specific quantitation of parasite loads (number of copies/µL of blood). The detection threshold was estimated at one amplicon copy per µL (approximately 20 parasites per mL) for *P. vivax* and 10^-3^ amplicon copies per µL (approximately 0.2 parasites per mL) for *P. falciparum.*

*Data analysis -* Data were analysed with STATA version 15.1 software (Stata, College Station, USA). Statistical significance was defined at the 5% level (two-tailed tests). Proportions were compared by applying standard Fisher or chi-square tests to contingency tables. Multiple logistic regression models were run to identify independent correlates of TaqMan-confirmed *P. vivax* infection in both surveys combined, while adjusting for potential confounders. Individual-level variables included in the model were age (categorised as 0-5, 6-15, 16-49 and ≥ 50 years), sex, bed net use past night (no; yes, untreated; yes, insecticide-treated), overnight stays in the forest in the last month for hunting or fishing (yes/no) and presence of any symptom within the past 7 days (yes/no) and presence of specific symptoms (no; mild; moderate; severe). The household-level variables were indoor residual spraying with insecticides within the past 6 months (yes/no) and wealth index (stratified into quartiles). Due to the nested structure of the data, we used the “melogit” STATA command to build mixed-effects logistic regression models that included the grouping variables as random factors. We had repeated observations for some participants (obtained during the 2018 and 2019 surveys; grouping variable, “survey”), who were clustered within households (grouping variable, “household”). Covariates were introduced in the models following a stepwise forward approach, and only those that were associated with the outcome at a significance level of at least 20% were retained in the final model. Participants with missing information were excluded from adjusted analysis, except for those with missing symptom information, for which a missing-information category was created. We next used a similar multiple logistic modelling strategy to test whether anaemia was significantly associated with TaqMan-diagnosed *P. vivax* infection, while adjusting for age (categorised as 0-5, 6-15, 16-49 and ≥ 50 years) and sex and excluding participants with *P. falciparum* infection diagnosed by microscopy or TaqMan assays. 

*Ethics -* The authors assert that all procedures contributing to this work comply with the ethical standards of the relevant national and institutional committees on human experimentation and with the Helsinki Declaration of 1975, as revised in 2008. Study protocols have been approved by the Institutional Review Board of the University Hospital of Universidade de São Paulo and by the National Committee of Ethics in Research, Brazilian Ministry of Health (CAAE number 64767416.6.0000.5467). Written informed consent was obtained from all study participants or their parents/guardians. 

## RESULTS

*Prevalence of malarial infection and disease -* Overall, 31.8 and 24% of the participants tested for malaria parasites in the 2018 and 2019 surveys, respectively, reported recent clinical signs or symptoms that might be associated with malaria. This information was missing for six participants in 2018 and eight participants in 2019. 

Table II shows the number of malarial infections, either symptomatic or not, diagnosed by conventional microscopy and species-specific, *cytb*-based TaqMan assays during the surveys. Only three infections were diagnosed by microscopy (two *P. falciparum* infections in the 2018 survey and one *P. vivax* infection in the 2019 survey). However, TaqMan assays detected 33 *P. vivax* infections (overall prevalence, 11.1%), 78.1% of which were asymptomatic. Interestingly, no additional *P. falciparum* infections missed by microscopy was diagnosed by molecular methods. Parasitaemia was typically very low, with a median of 1.7 amplicon copy per µL (interquartile range, 1 to 7.8 copies per µL). The highest parasitaemia was 12,143 copies per µL (corresponding to 243 parasites/µL), found in the only study participant with microscopy-positive *P. vivax* infection - an 8 years-old male child who lived in Barro Vermelho and reported eight past malaria episodes and no recent travel. This was also the only participant with a *P. vivax* density above the detection threshold of field microscopy, estimated at 100 parasites per µL,^22^ corresponding approximately to 5,000 *cytb* gene copies per µL. 

**TABLE II:** Number of *Plasmodium falciparum* and *Plasmodium vivax* infections diagnosed by microscopy and TaqMan assays according to the presence or absence of malaria-related symptoms during two cross-sectional surveys in villages along the Azul River, upper Juruá Valley, Acre state, Brazil, 2018 and 2019

		2018 survey (% positive)		2019 survey (% positive)	
Symptoms	Species^*a*^	Microscopy	TaqMan	Microscopy	TaqMan
Yes	*P. falciparum*	1 (2.1)	1 (2.2)	0 (0.0)	0 (0.0)
	*P. vivax*	0 (0.0)	3 (6.5)	1 (2.8)	4 (11.1)
	Tested (n)	47	46	36	36
No	*P. falciparum*	1 (1.0)	1 (1.0)	0 (0.0)	0 (0.0)
	*P. vivax*	0 (0.0)	12 (12.9)	0 (0.0)	13 (12.3)
	Tested (n)	95	93	106	106
	Missing symptom information	6	6	8	8
Total	*P. falciparum*	2 (1.3)	2 (1.4)	0 (0.0)	0 (0.0)
	*P. vivax*	0 (0.0)	16 (11.0)	1 (0.7)	17 (11.3)
	Tested (n)	148	145	150	150

*a*: no mixed-species (*P. falciparum* + *P. vivax*) infection was diagnosed by microscopy or TaqMan assays.

Similar proportions of participants who reported (8.5%; 7/82) or did not report recent clinical symptoms (12.6%; 25/199) had a *P. vivax* infection diagnosed by TaqMan, with Yates corrected chi-square = 0.576, 2 degrees of freedom (d.f.), p = 0.448 (missing information for 14 participants). Only three of the 32 *P. vivax-*infected participants with complete information reported recent fever (two mild, one moderate), none reported chills and five reported headache (three mild, two intense) (missing information for one participant). Other symptoms were rarely reported. A single participant, the one with *P. vivax* infection diagnosed by both microscopy and TaqMan during the 2019 survey, had a full-blown malaria paroxysm at the time of enrolment. 

The proportions of infections that were symptomatic vs. asymptomatic did not vary significantly with age: these were 0 vs. 2 (0%) in the 0-5 years age group, 2 vs.7 (22.2%) in 6-15 years age group, 4 vs. 13 (23.5%) in the 16-49 years age group and 1 vs. 3 (25%) in the ≥ 50 years age group (Fisher exact test, 3 d.f., p = 1.000; 32 participants with complete data). Moreover, the mean age of participants with symptomatic (26.0 years) and asymptomatic (27.4 years) *P. vivax* infections was similar (*t* = 0.84, p = 0.402; 32 participants with complete information).

The relative proportions of positive vs. negative TaqMan assay results for *P. vivax* did not vary significantly across villages: 7 vs. 62 in Três Unidos, 1 vs. 1 in Valparaíso, 3 vs. 29 in Buritis, 0 vs. 7 in Belo Horizonte, 4 vs. 36 in Nova Lição, 5 vs. 14 in Barro Vermelho, 10 vs. 84 in Bom Sossego, 0 vs. 7 in Bela Vista, 3 vs. 21 in Bom Jesus and 0 vs. 1 in Queimadas (chi-square = 9.607, 9 d.f., p = 0.383).

Haemoglobin measurements were available for 262 participants who were screened for malaria. We found no significant association between anaemia and *P. vivax* positivity by TaqMan assays, after adjusting for age and sex, with an adjusted odds ratio (OR) of 0.59 and a 95% confidence interval (95%CI) of 0.27-1.29, p *=* 0.136 (n = 260 after excluding two *P. falciparum*-infected participants). 

*Correlates of P. vivax infection -* We next sought to identify independent correlates of TaqMan-diagnosed *P. vivax* infection by using multivariable analysis (Table III). Interestingly, *P. vivax* infection was positively associated with age (p = 0.019). Other significant predictors of being infected were male sex (possibly due to occupational exposure associated with farming, fishing or hunting^7^) and use of an insecticide-treated bed net past night (possibly because of the users´ perception of their increased risk of infection^23^). Moreover, participants reporting one or more recent symptoms were significantly less likely to be infected with *P. vivax*, compared with asymptomatic participants (OR 0.52; p = 0.038), after adjusting for age, sex and bed net use (Table III). 

**TABLE III:** Correlates of *Plasmodium vivax* infection in villages along the River Azul, upper Juruá Valley, Acre state, Brazil

		Unadjusted analysis			Multivariable analysis^*a*^		
	Positive (n)/						
Variable	Tested^*b*^ (n)	OR	(95%CI)	p-value	aOR	(95%CI)	p-value
Age (years)							
0-5	2/35	1			1		
6-15	9/104	1.64	(0.14-1.92)	0.695	1.67	(0.08-34.54)	0.737
16-49	17/131	2.42	(0.54-15.0)	0.215	2.76	(0.33-22.81)	0.346
≥ 50	4/23	3.93	(0.27-57.10)	0.316	5.25	(0.12-223.52)	0.386
				p for trend = 0.001			p for trend = 0.019
Sex							
Female	15/159	1			1		
Male	18/136	1.65	(1.51-1.81)	< 0.0001	1.87	(1.03-3.38)	0.040
Bed net use past night							
No	3/33	1			1		
Yes, insecticide-untreated	8/108	0.91	(0.73-1.12)	0.376	0.84	(0.46-1.53)	0.574
Yes, insecticide-treated	21/151	2.09	(1.91-2.28)	< 0.0001	2.05	(1.03-4.10)	0.040
Any malaria-related symptom within the past 7 days							
No	25/199	1			1		
Yes	7/82	0.61	(0.33-1.12)	0.111	0.52	(0.28-0.96)	0.038
Wealth index quartile							
1 (poorest)	9/77	1			1		
2	7/80	0.69	(0.17-2.71)	0.591	0.56	(0.36-0.86)	0.008
3	11/77	1.32	(0.61-2.86)	0.483	0.73	(0.13-3.99)	0.718
4 (least poor)	6/61	0.82	(0.21-3.22)	0.774	0.78	(0.08-7.45)	0.829
				p for trend = 0.070			p for trend = 0.902

*a*: adjusted for all variables listed in the table by using mixed-effects multiple logistic regression analysis (n = 291 observations with complete information; seven observations were excluded due to lacking data for selected variables); *b*: numbers refer to observations, not individuals, as some individuals were tested during both surveys (i.e., contributed two observations). Totals differ for some variables because of missing information. 95%CI: 95% confidence interval; aOR: adjusted odds ratio; OR: odds ratio.

## DISCUSSION

This study provides further evidence that clinically silent *P. vivax* infections remain relatively common in remote riverine villages in the Amazon.^4^ ^-^ ^8^ This finding has clear public health implications, since asymptomatic carriers of *P. vivax* are estimated to contribute 28.2 to 79.2% of all *An. darlingi* infections with this parasite species across the region.^14^ Importantly, asymptomatic villagers can move parasites across the rural-urban interface and originate outbreaks in more densely populated urbanised spaces.^24^ ^)^ To test this hypothesis, we are currently analysing the genetic connectivity between malaria parasites that circulate in rural and urban areas of the Juruá Valley.

Why have *P. vivax* infections persisted while *P. falciparum* infections are now infrequent in riverine villages? *P. vivax* may be less susceptible than *P. falciparum* to the malaria elimination strategies implemented in Brazil over the past 2 decades, which are focused on early microscopic diagnosis and prompt treatment of clinically apparent infections.^25^ First, *P. vivax* infections are often overlooked by routine microscopy and antigen-based rapid diagnostic tests because of their low parasitaemia,^26^ which results from the strict tropism of blood-stage parasites for reticulocytes - since only 0.5 to 1.5% of circulating red blood cells in healthy adults.^27^ Second, mature gametocytes are found in the peripheral blood early in the course of *P. vivax* infections, often before the laboratory diagnosis can be confirmed and antimalarial therapy is administered.^25^ In consequence, *P. vivax-*infected individuals are likely to infect mosquitoes even when prompt diagnosis and treatment are widely available. Third, *P. vivax* sporozoites originate hypnozoites, dormant liver stages that can cause one or more relapses several weeks or months after the primary infection.^28^ In the Amazon, 11% of *P. vivax* infections are estimated to relapse within 12 months, despite routine prescription of 3.5 mg/kg of primaquine over 7 days.^29^ ^)^ Therefore, high-dose primaquine regimens (e.g., 7.0 mg/kg of primaquine over 14 days) may be required to eradicate hypnozoites in this region.^30^ Parasite resistance to schizontocidal antimalarials, leading to blood-stage recrudescence shortly after treatment, is unlikely to contribute significantly to the overall vivax malaria burden in the Juruá Valley hotspot of Brazil, where the standard chloroquine-primaquine therapy remains highly efficacious.^31^ ^,^ ^32^

Some of our current findings are at odds with previous observations suggestive of strong antimalarial immunity among rural Amazonians.^4^ ^,^ ^6^ ^,^ ^9^ ^)^ First, we found a trend towards “*increased*” (instead of “*decreased*”) prevalence of *P. vivax* infection with increasing age (Table III). Such a trend is typically seen in non-immune migrants who have recently arrived in frontier farming settlements, where malaria affects mostly adult males and under-five children are usually spared.^33^ ^,^ ^34^ ^)^ However, this is “*not*” the typical age-related prevalence gradient found among native Amazonians living in traditional villages, who are exposed to malaria since birth and become gradually less susceptible to infection.^6^ ^,^ ^13^ ^,^ ^14^ We suggest that adults from the Azul River area did not develop significant anti-parasite immunity despite their life-long exposure to infection. Second, the vast majority of these infections were asymptomatic in all age groups. The proportion of infections that were asymptomatic was similar across age groups, indicating that adults did not develop more efficient anti-disease or clinical immunity than children.^4^ ^,^ ^6^ Therefore, adults were more likely than children to be infected with *P. vivax*, but as likely as children to have symptoms once infected.

We hypothesise that low parasite virulence, in addition to acquired immunity, might also contribute to the high proportion of asymptomatic *P. vivax* infections found in children and adults from isolated villages along Azul River. Virulent parasites are more likely to survive the within-host competition between co-infecting clones in high-transmission settings, where super- or co-infection events are common. In contrast, nonvirulent lineages may have a selective advantage in remote low-transmission settings, where parasite diversity is low due to the limited gene flow between villages and hosts are more likely to harbour single-clone asymptomatic infections.^35^ ^,^ ^36^ Long-lasting infections with low-virulence parasites, with no co-infecting lineages competing for limited host resources,^36^ are expected to remain undiagnosed and untreated and thus contribute to transmission over several weeks.^12^ ^-^ ^14^ Although most support for the “virulence hypothesis” arises from epidemiological studies in *P. falciparum*-dominated areas,^36^ we speculate that within-host competition may also select for relatively more *P. vivax* strains in co-infections with two or more lineages.^37^ ^)^ Larger studies with molecular analysis of the multiplicity of *P. vivax* infection are clearly needed to test this hypothesis in the Amazon and other settings.

Alternatively, human genetics, in addition to restricted reticulocyte tropism, may also contribute to limit parasite growth across all age groups. The risk of presenting symptoms is directly (but not linearly) proportional to blood-stage parasite density in *P. vivax* infections and carriers with very low parasitaemia are expected to remain asymptomatic.^12^ Our study participants were not tested for well-known *P. vivax* resistance factors, such as the Duffy-negative (Fy) phenotype.^38^ Interestingly, Duffy negativity appears to be substantially less frequent in the Amazon (e.g., 6.4% in the study of Barbosa et al.^9^), where malaria is endemic, than in Brazil as a whole (estimated at 13.2% by King et al.^39^). However, partial resistance to blood-stage *P. vivax* infection may also be conferred by the Fya polymorphism,^39^ which is substantially more prevalent in the region,^9^ ^,^ ^39^ as well as by other genetic factors yet to be determined.

The present study has some limitations. First, we screened a relatively small population sample for malaria parasites. Participants may not be representative of the entire population of Azul River villages. Logistic limitations prevented house-to-house visits to be carried out in the smallest and most remote villages in the region. Second, individuals with current illnesses, either infectious or not, were overrepresented in our population sample, as they were more likely to seek health care during the field surveys. Third, we had no detailed information on key individual and contextual predictors of malaria risk, such as occupation and behaviour, cumulative exposure to infection and housing quality (e.g., housing material). Fourth, as both cross-sectional surveys were carried out at the end of the rainy season, we could not estimate the seasonal variation in malaria prevalence. Finally, the infrequency of *P. falciparum* infections precluded between-species comparisons of malaria risk factors in the study population.

We conclude that *P. vivax* infections remain common, but are substantially underdiagnosed by microscopy in riverine villages in the main malaria transmission hotspot of Brazil. The vast majority of infections are asymptomatic, due to either clinical immunity or low parasite virulence. Periodic active case detection is required to identify parasite carriers who remain asymptomatic and are likely to fuel residual malaria transmission in this and other similar hard-to-reach communities across the Amazon.
